# Communication Tools for the Modern Doctor Bag. Physician Patient Communication Part 1: Beginning of a medical interview

**DOI:** 10.3402/jchimp.v1i3.8428

**Published:** 2011-10-17

**Authors:** Sapna Patel Kuehl

**Affiliations:** Department of Internal Medicine, St. Agnes Hospital, Baltimore, Maryland

**Keywords:** Physician patient communication, communication skills, communicating with patients

## Abstract

Effective physician patient communication is essential to best practice in medicine. Good communication with patients is critical in making the right diagnosis, improving compliance and overall outcomes for our patients (as well as improving physician satisfaction.) Communication skills can be learned and need to be taught, practiced and given the same emphasis as other core competencies in medicine. The focus of this article is on the Calgary-Cambridge Model for physician patient communication in the context of a medical interview. The beginning of a patient encounter is discussed, with emphasis on appropriate introductions and attentive active listening.

The wisdom of Thomas Aquinas, the logic of Newman, and the clinical genius of Osler will not be effective in making well a patient who does not fully understand why he is sick, or what he must do to get well. A first rate clinician trains himself to do two things exceedingly well: to talk to his or her patients and to listen to them. And he acts similarly with responsible family members.

Dr. Philip Tumulty, MD, *What is a Clinician and What Does He Do?* (1)

Communication is at the heart of what we do as physicians. Not only is it the most basic and powerful of all the skills we possess, but it is also essential to our joy and satisfaction as healers. An average physician conducts more than 200,000 consultations in their professional career (2).

Our patient consultations and interviews are the crux of what we do, and becoming more effective at them improves our ability to be good clinicians. Effective physician–patient communication drives efficient and accurate diagnosis. Sir William Osler is known for saying: listen to your patient, he is telling you the diagnosis. Our ability to communicate well improves diagnostic abilities by focusing on the correct diagnosis sooner, contributing to a reduction in unnecessary costly and oftentimes invasive tests (1, 2). Unfortunately, the ‘time with our patients’ is increasingly threatened by multiple factors, including financial pressures, medical complexity, and numerous regulatory requirements. These limitations can result in decreased job satisfaction and increased risk for burnout and cynicism among clinicians.

Excellent communication skills provide us with the capacity to improve the quality and efficiency of the clinical care we can provide, while simultaneously making us more valuable team leaders and team members in healthcare (2, 3). In addition to the shortened time to reach diagnosis and the resulting decreased resource utilization, there is an important business benefit achieved with improved communication (3). Patient turnover in a practice can be a significant problem. It can cost seven times more to get a new patient than to keep an old one (4). Greater patient loyalty and satisfaction translates to greater patient retention, free advertising of the practice, and increased practice success overall. The strongest benefit publicized in relevant literature is the idea that effective and quality communication reduce the risk of malpractice incidences (5); communication problems between patients and physicians were identified in 70% of malpractice depositions reviewed (3, 6). Primary care physicians with no malpractice claims are more likely to use better communication approaches than the physicians who had a history of malpractice claims, including checking for understanding and encouraging patients to talk more frequently (7). In addition, communication is in the top three of the most frequently identified root causes for sentinel events as reviewed by the Joint Commission in 2008–2010 (8).

Good communication skills need to be taught, practiced, and given the same emphasis as other core skills in medicine. Getting better at communication is a lifelong journey, with continuous active improvement required to reach excellence. The Accreditation Council of Graduate Medical Education (ACGME) recognized communication and interpersonal skills as a core competence for training of physicians in 2001 (9). The Joint Commission has also recognized the importance of effective communication to providing quality healthcare, and they recently published a roadmap to inspire hospitals to integrate communication, cultural competence, and patient-and family-centered care fields into their organizations (10).

There are many different physician–patient communication models in the literature, and many have been shown to be effective in promoting behavior change among clinicians. The focus of this article series is on elaborating on the Calgary-Cambridge Guide, and on the ideas discussed in the books by Silverman, Kurtz and Draper – *Skills for Communicating with Patients* and its companion volume, *Teaching and Learning Communication Skills in Medicine* (11). In this article series, the skills of physician–patient communication will be explored further in the context of a medical interview. This article specifically focuses on the initial part of the patient encounter.

## Beginning of the interview: introduction and listening

The beginning of an interview is a powerful time to make important first impressions, establish rapport, and identify problems that set the tone for the entire relationship (2). Studies conducted in the primary care field suggest evidence that at least half of a patient's complaints are not elucidated during an interview (12). Moreover, doctor and patient disagreed on the main presenting problem in up to 50% of visits (13)!

Preparation prior to the interview is important. As Silverman et al emphasize, it is not uncommon to be mentally focusing on the last task or our last patient, or perhaps thinking of our own personal needs, creating a situation such that our attention is not fully focused on the upcoming new patient (2). Distraction hampers effective communication. Our body language often betrays our distraction, and may set the wrong impression. We must allow ourselves to be free to concentrate on our upcoming patient (2). Stopping to take a deep breath before knocking may allow us to recenter ourselves on the upcoming encounter and give it our undivided attention (3). The simple act of waiting after knocking instead of knocking and then entering immediately is a common courtesy, which although it delays an additional second, it sets a tone of respect (3).

Greeting the patient, making introductions, and clarifying your role as a caregiver can be incredibly helpful in new encounters. This is especially beneficial in the in-patient setting, where multiple caregivers are involved and each one's role is not as clear. ‘Hello, Mrs. Williams, I'm Dr. Jones. May I sit here?’ or ‘Hello, Mr. Green, I'm Dr. Smith. I will be the senior doctor seeing you during your stay in the hospital. Can I spend a few minutes with you now discussing your problems and examining you?’ Our body language, behavior, and demeanor can be vital in showing respect and interest in the patient's condition (2). As most of us are more comfortable speaking while sitting up, unless the medical condition dictates otherwise, helping the patient to sit up and looking out for their comfort before we begin the interview is helpful as well (2). When possible, the act of sitting down at eye level with a patient helps increase our patient's comfort. Sitting down gives the important impression that one is not rushed and is willing to devote their time and full attention to the patient (2).

Human beings love the sound of their own name (3). It is believed to be the most important word for each individual. At a minimum, clinicians must use the patient's name at the beginning and the end of the visit (3). Using a patient's name avoids errors and is a sure way to clarify that you are with the right patient. In addition, it indicates your respect for that particular individual. Studies tell us that right after you use a person's name you have that person's complete attention for the next 30 seconds (3).

The opening question should be open ended and essentially convey our interest in listening. ‘How can I help you?,’ ‘Tell me what you have come to see me about.’, or ‘Tell me why you are in the hospital?’ are all more specific than ‘How are you doing?’, but they all have their place depending on the context of the encounter. One of the challenges physicians face is with active listening. The average physician allows a patient to talk for about 20–30 seconds before interrupting (14). Interestingly, if allowed to talk uninterrupted, most patients will stop talking in less than two minutes, with a mean time of 92 seconds, and the median was a mere 59 seconds (14)! Further, only 2% of patients talked for greater than five minutes, and in all cases the physicians considered the information they were given to be relevant (14). More importantly, jumping too quickly into closed and directed questioning mode may lead to other challenges (2). In a study of internal medicine residents and physicians in primary care, Beckman and Frankel showed that while patients do not present their problems in the order of clinical importance, doctors often assume erroneously that the first complaint mentioned is the only one that the patient brought (15).

Byrne and Long showed that doctors usually have fixed routines for interviewing patients that demonstrate little capacity for variation to meet a patient's individual needs (16). Beckman and Frankel further found that doctors’ words, questions, and tones can easily and inadvertently direct the patient away from disclosing their reasons that brought them to see the doctor (15). Additionally, the early pursuit of closed questioning may not only prevent doctors from discovering all the issues that a patient wishes to discuss but also can lead one down the wrong path to an incorrect diagnosis and create inefficiencies in history taking (2).

## Attentive listening

Attentive listening is more than just physical presence – it requires focus, concentration, and active involvement. Proper body language is important, as patients will pick up on both verbal and non-verbal cues, and our responses (2). There are four specific skill areas that can help us to develop our ability to listen attentively, namely (1) the wait time, (2) facilitative response, (3) non-verbal skills, and (4) picking up on verbal and non-verbal cues (2). The wait time defines the shift from speaking to listening. Studies of non-medical teachers by Rowe found that if teachers were trained to increase their pauses to three seconds between key points, big changes occurred in student behavior in classes (17). The students contributed more often, spoke for longer, asked more questions, and showed more evidence for their thinking (17). Wait time in physician–patient interviews similarly gives the patient time to think and to contribute more without interruption. Facilitative response skills such as repetition, paraphrasing, and interpretation are very helpful in later stages of the interview, but using a more neutral facilitative phrase like ‘uh-huh’ or ‘go on’ are much more helpful early on in the interview and also less distracting (2). However, poor body language (closed stance, poor eye contact, etc.) can definitely override verbal messages.

## Conclusion

So far, we have explored the beginning portion of a medical interview. By establishing a strong rapport and gaining a patient's trust and respect the clinician is much more likely to obtain an accurate and efficient history, while at the same time provide a supportive and comfortable environment for the patient. This is the basis of a collaborative partnership between the clinician and the patient, and allows the goals of accuracy, efficiency, and supportiveness in medical communication to be achieved (2). This critical portion of the interview paves the way to effective and efficient data gathering, relationship building, and plan of care discussions. A future article will focus on effective strategies to gather data, set agenda, discuss care plans, and efficiently close interviews. A shared decision-making model will be explored with relationship-building strategies and other tips on effective verbal and non-verbal communication.
